# Internet-based psychodynamic versus cognitive behaviour therapy for adolescents with depression: study protocol for a non-inferiority randomized controlled trial (the ERiCA study)

**DOI:** 10.1186/s13063-020-04491-z

**Published:** 2020-06-29

**Authors:** Jakob Mechler, Karin Lindqvist, Per Carlbring, Peter Lilliengren, Fredrik Falkenström, Gerhard Andersson, Naira Topooco, Robert Johansson, Nick Midgley, Julian Edbrooke-Childs, Hanne-Sofie J. Dahl, Rolf Sandell, Agneta Thorén, Randi Ulberg, Katja Lindert Bergsten, Björn Philips

**Affiliations:** 1 Department of Psychology, Stockholm University, Stockholm, Sweden; 2 Ersta Sköndal Bräcke University College, Stockholm, Sweden; 3 Department of Behavioural Sciences and Learning, Linköping University, Linköping, Sweden; 4 Department of Clinical Neuroscience, Karolinska Institute, Stockholm, Sweden; 5 Center for m2Health, Palo Alto, CA USA; 6 Child Attachment and Psychological Therapies Research Unit (ChAPTRe), Anna Freud Centre, London, UK; 7 Department of Clinical, Educational and Health Psychology, University College London, London, UK; 8 Evidence Based Practice Unit, Anna Freud National Centre for Children and Families, London, UK; 9 Vestfold Hospital Trust, Tønsberg, Norway; 10 Department of Psychology, University of Oslo, Oslo, Norway; 11 Department of Psychology, Lund University, Lund, Sweden; 12 The Erica Foundation, Stockholm, Sweden; 13 Division of Mental Health and Addiction, University of Oslo, Oslo, Norway; 14 Department of Psychology, Uppsala University, Uppsala, Sweden; 15 Department of Department of Psychiatric Research, Department of Adult Psychiatry, Diakonhjemmet Hospital, Oslo, Norway

**Keywords:** Depression, Non-inferiority trial, Adolescents, Psychodynamic, CBT, Internet-based treatment

## Abstract

**Background:**

Adolescent depression is a common mental health problem and there is an urgent need for effective and accessible treatments. Internet-based interventions solve many obstacles for seeking and receiving treatment, thus increasing access to effective treatments. Internet-based cognitive behavioural therapy (ICBT) for adolescent depression has demonstrated efficacy in previous trials. In order to broaden the range of evidence-based treatments for young people, we evaluated a newly developed affect-focused Internet-based psychodynamic treatment (IPDT) in a previous study with promising results. The purpose of the planned study is to evaluate the efficacy of IPDT for adolescent depression in a non-inferiority trial, comparing it to ICBT.

**Methods:**

The study will employ a parallel randomized non-inferiority design (ratio 1:1; *n* = 270). Eligible participants are adolescents 15–19 years suffering from depression. The primary hypothesis is that IPDT will be non-inferior to ICBT in reducing depressive symptoms from pre-treatment to end of treatment. Secondary research questions include comparing outcomes of IPDT and ICBT regarding anxiety symptoms, emotion regulation and self-compassion. Additional data will be collected to evaluate cost-effectiveness as well as investigating predictors, moderators and mediators of outcome. In addition, we will examine long-term outcome up to 1 year after end of treatment. Diagnostic interviews with MINI 7.0 will be used to establish primary diagnosis of depression as well as ruling out any exclusion criteria. Both treatments consist of eight modules over 10 weeks, complemented with therapist support through text messages and weekly chat sessions. Primary outcome measure is the Quick Inventory of Depressive Symptomatology in Adolescents Self-Rated (QIDS-A17-SR). Primary outcome will be analysed using data from all participants entering the study using a multilevel growth curve strategy based on the weekly measurements of QIDS-A17-SR. The non-inferiority margin is defined as *d* = 0.30.

**Discussion:**

This trial will demonstrate whether IPDT is non-inferior to ICBT in the treatment of adolescent depression. The study might therefore broaden the range of evidence-based treatment alternatives for young people struggling with depression. Further analyses of data from this trial may increase our knowledge about “what works for whom” and the pathways of change for two distinct types of interventions.

**Trial registration:**

ISRCTN12552584, Registered on 13 August 2019.

## Administrative information

Note: the numbers in curly brackets in this protocol refer to SPIRIT checklist item numbers. The order of the items has been modified to group similar items (see http://www.equator-network.org/reporting-guidelines/spirit-2013-statement-defining-standard-protocol-items-for-clinical-trials/).

**Table:** 

Title {1}	Internet-based psychodynamic therapy versus cognitive behaviour therapy for adolescents with depression: Study protocol for a non-inferiority randomized controlled trial (the ERiCA study)
Trial registration {2a and 2b}.	The trial was prospectively registered in the World Health Organization’s International Clinical Trials Registry Platform (ISRCTN, identification number 12552584). Registered 13 August 2019, https://www.isrctn.com/ISRCTN12552584
Protocol version {3}	2/18/20, version 1 5/20/20, version 2
Funding {4}	The project is co-funded by the Kavli Trust (grant no. 32/18) and the Department of Psychology, Stockholm University.
Author details {5a}	Jakob Mechler, MSc, Department of Psychology, Stockholm University, Stockholm, Sweden, jakob.mechler@psychology.su.se Karin Lindqvist, MSc, Department of Psychology, Stockholm University, Stockholm, Sweden, karin.lindqvist@psychology.su.se, Per Carlbring, Ph.D., Department of Psychology, Stockholm University, Stockholm, Sweden, per.carlbring@psychology.su.se Peter Lilliengren, PhD, Ersta Sköndal Bräcke University College, Stockholm, Sweden, peter.lilliengren@esh.se Fredrik Falkenström, PhD, Department of Behavioural Sciences and Learning, Linköping University, Linköping, Sweden, fredrik.falkenstrom@liu.se Gerhard Andersson, PhD, Department of Behavioural Sciences and Learning, Linköping University, Linköping, Sweden; Department of Clinical Neuroscience, Karolinska Institute, Stockholm, Sweden, gerhard.andersson@liu.se Naira Topooco, PhD, Department of Behavioural Sciences and Learning, Linköping University, Linköping, Sweden; Center for m^2^Health, Palo Alto, CA, United States, Naira.topooco@liu.se Robert Johansson, PhD, Department of Psychology, Stockholm University, Stockholm, Sweden, robert.johansson@psychology.su.se Nick Midgley, PsychD PhD, Child Attachment and Psychological Therapies Research Unit (ChAPTRe), Anna Freud National Centre for Children and Families/University College London, UK, nick.midgley@annafreud.org Julian Edbrooke-Childs, PhD, Department of Clinical, Educational and Health Psychology, University College London; Evidence Based Practice Unit, Anna Freud National Centre for Children and Families, London, UK, Julian.Childs@annafreud.org Hanne-Sofie J Dahl, PhD, Vestfold Hospital Trust; Department of Psychology, University of Oslo, h.s.j.dahl@psykologi.uio.no Rolf Sandell, PhD, Department of Psychology, Lund University, Lund, Sweden, rolf.sandell@psy.lu.se Agneta Thorén, PhD, The Erica Foundation, Stockholm, Sweden, agneta.thoren@ericastiftelsen.se Randi Ulberg, PhD MD, University of Oslo, Oslo, Norway, randi.ulberg@medisin.uio.no Katja Lindert Bergsten, PhD, Department of Psychology, Uppsala University, Uppsala, Sweden, katja.bergsten@psyk.uu.se Björn Philips, PhD, Department of Psychology, Stockholm University, Stockholm, Sweden, Bjorn.philips@psychology.su.se (corresponding author)
Name and contact information for the trial sponsor {5b}	Department of Psychology, Stockholm University, SE-106 91 Stockholm, Sweden
Role of sponsor {5c}	The study PI, the project coordinators and several other members of the research group are employed by the sponsor Department of Psychology, Stockholm University. The PI and project coordinators are responsible for collection and management of data. The entire research group will be involved in analysis and interpretation of data, as well as writing of the report. The PI makes the decision to submit the report for publication, and all authors will approve the submitted manuscript. The co-funders, the Kavli Trust, has no part in study design, collection, management, analysis/interpretation of data, writing of the report or decision to submit the report for publication.

## Introduction

### Background and rationale {6a}

Research suggests that half of all mental disorders have their onset prior to 14 years of age [1]. In childhood, anxiety and depression seem to be the most common diagnoses [2]. According to recent longitudinal data, the lifetime prevalence of adolescent depression is 11.4%, accompanied by significantly higher risks of adversity through adolescence and adulthood, such as recurrent depression, other mental health issues, lower educational attainment, and relational problems [3]. Depression in adolescence is associated with a five-fold risk of suicide attempts, a two-fold risk of later depression, increased use of psychiatric and medical care, as well as decreased functioning in school, work, family, and social life [4]. Data indicates that merely 13.3% of children with emotional disorders (without comorbid attention-deficity-hyperactivity disorder or behavioural disorders) have been in contact with mental health services [5]. It is therefore of the utmost importance to provide early and preventive interventions for these disorders, which can reach young people who may not otherwise be seen in specialist mental health services.

Accumulating evidence suggests that the effects of psychological interventions delivered via the Internet are comparable to traditional face-to-face treatments [6]. In a meta-analysis of Internet-based interventions for children and adolescents, eleven studies were identified that target psychiatric problems, demonstrating moderate to large effects comparable to those observed in face-to-face psychotherapies [7]. However, the studies’ quality varied substantially, highlighting the need for new high-quality studies in the field [8]. The efficacy of Internet-based cognitive behavioural therapy (ICBT) with added chat sessions for depressed adolescents has been evaluated in two randomized controlled trials (RCTs). In both studies, ICBT was superior to attention control [9, 10].

Cognitive behavior therapy (CBT) and pharmacological treatments are the most established and often recommended treatments for depressed adolescents in many treatment guidelines. However, adverse effects have been linked with pharmacological treatments, including the elevated risk of suicidality [11]. CBT has been extensively researched with robust and reliable effects, but a substantial proportion of adult patients suffering from depression do not gain enough benefit from this form of psychotherapy [12]. Meta-analyses regarding adolescent depression depict a response rate to face-to-face CBT of about 60% [13, 14], and ICBT for mixed disorders in childhood and adolescence between 20% and 76% [7]. Although CBT and ICBT are effective, the significant number of non-responders, along with the factor that offering choice and shared decision-making is an important part of effective health provision, poses two urgent issues to psychotherapy research: (1) the finding of new treatment methods that can complement and/or serve as alternatives to current treatments to effectively treat more patients and (2) the development of methods of detecting non-responders, dropouts, and patients that may deteriorate during treatment (c.f [15].) to enhance, adapt, or alter treatment.

In order to broaden the range of evidence-based therapies available to young people, provide meaningful choice and identify effective treatments for depressed adolescents, more research should focus on treatments that differ from CBT, including psychodynamic psychotherapy (PDT). A meta-analysis of short-term PDT [16] for children and adolescents reported large within-group effects across several outcome domains, with medium to large effects regarding anxiety and mood with continued gains during follow-up; no differences were identified in effectiveness compared to other psychotherapies. These findings have been corroborated by a recent large RCT, where PDT was found to be equally as effective as CBT in the treatment of youth depression [17]. To date, five randomized studies of Internet-based interventions grounded in psychodynamic theory (IPDT) have been published, all of which address adults with mood or anxiety disorders and report medium to large effects compared to control conditions [18–22]. 

### Objectives {7}

This study protocol describes a non-inferiority RCT that will evaluate the efficacy of therapist-assisted ICBT and IPDT for adolescent depression. The study has been preceded by a pilot RCT (*n* = 76) that investigated the efficacy of a novel IPDT treatment for depressed adolescents compared to control condition consisting of weekly symptom follow-up and online supportive contact [23]. The pilot trial showed clinically and statistically significant effects of IPDT compared to control thereby depicting the treatment’s strong acceptability and feasibility. Answering to the need of increased accessibility to digital health interventions, the main goal of the planned study is to further compare the IPDT treatment for depressed adolescents to an established evidence-based treatment (ICBT) in terms of efficacy, cost-effectiveness and factors that affect differential suitability. The primary aim is to examine whether or not therapist-assisted IPDT is non-inferior to therapist-assisted ICBT in treating depression.

#### Primary research question/primary outcome

Is IPDT non-inferior to ICBT in reducing depressive symptoms from pre-treatment to end of treatment in adolescents?

#### Secondary research questions/secondary outcomes

To compare the outcomes of IPDT and ICBT regarding anxiety reduction, emotion regulation and self-compassion.

Data will additionally be collected to make the following secondary analyses in further papers: (1) evaluate and compare cost-effectiveness, (2) identify predictors of outcome, (3) investigate moderators of outcome, (4) examine possible outcome mediators and (5) investigate long term outcome up to 1 year after end of treatment.

### Trial design {8}

The design is a non-inferiority parallel group RCT (*n* = 270). IPDT will be compared to ICBT for adolescents aged 15–19 years with major depressive disorder (MDD). Specific inclusion and exclusion criteria are described under the eligibility criteria. The non-inferiority design tests whether or not IPDT is no less efficacious than ICBT. Eligible participants will be randomized to one of the two arms (1:1 ratio). This non-inferiority RCT will be conducted from 2019 to 2022.

## Methods: participants, interventions and outcomes

### Study setting {9}

The project is based at Stockholm University, Sweden, in close collaboration with Linköping University. As treatment is conducted over the Internet, we will be able to recruit participants across Sweden, which will allow us opportunities for recruiting a larger and more heterogeneous sample regarding several aspects, such as geographic location and socioeconomic status [24]. The project will apply the well-developed infrastructure and the secure and responsive Internet platform developed especially for studies of Internet-based treatments at Linköping University [25].

### Eligibility criteria {10}

Adolescents 15–19 years who have a primary diagnosis of MDD according to DSM-5 [26] are eligible for inclusion. Participants must have access to a computer/smartphone/tablet with Internet connection and be able to read, write, and speak Swedish without the aid of an interpreter. Exclusion criteria include substantial risk of suicide (mainly indicated by clear intent and/or plans reported in the Columbia-Suicide Severity Rating Scale (C-SSRS [27]) interview) and/or earlier suicide attempts, current participation in other psychological treatment, psychotropic medication not stable the last month (or with planned dose adjustments), primary diagnoses other than MDD or current fulfilment of any of the following diagnoses: any psychotic disorder, bipolar I/II disorder, antisocial personality disorder or autism spectrum disorder. Comorbid drug or alcohol abuse is also set as exclusion criteria, whilst withdrawal criteria shall encompass patients who deteriorate such that they become suicidal; these individuals will be withdrawn from treatment and referred to psychiatric care.

The primary depression diagnosis will be established at baseline through telephone interviews using the MINI 7.0 [28]. The MINI can be administered in a short period of time, for which clinical interviewers solely require brief training. Further, suicidality will be assessed with the C-SSRS [27].

#### Therapists

The therapists will be master students recruited from a Swedish clinical programme in psychology (300 credits) in their final phase of psychologist training when they attend their psychotherapy courses. Students who have chosen CBT courses will be recruited as ICBT therapists, whilst students who have chosen PDT courses will be recruited as IPDT therapists. Therapists in the project will be trained in one approach of Internet treatment and supervised weekly by experienced psychotherapists in their respective modalities.

### Who will take informed consent? {26a}

Written informed consent is collected initially and the participants subsequently confirm their consent at two additional occasions. First, participants shall provide their informed written consent in the online application forms prior to screening. Next, participants are given repeated oral study information in the beginning of the diagnostic telephone interview, in which they are encouraged to ask questions, and again asked for consent. The telephone interviewers also ask some follow-up questions to ensure that the participants have understood the basic procedures of the study. Furthermore, after decision to include a participant, prior to randomization, a message is sent to the participant over the secure Internet platform to offer participation in the study, and the participants have to send a reply that confirms their consent to participate. According to Swedish law, parental consent is not needed if the adolescent is ≥ 15 years old, given that the adolescent is able to understand the study information and what participation in the study would mean. Therefore, participants are urged to tell their parents about partaking in the study, but parental consent is not mandatory.

### Additional consent provisions for collection and use of participant data and biological specimens {26b}

No ancillary studies will be undertaken and no biological specimens will be collected.

## Interventions

### Explanation for the choice of comparators {6b}

Internet-based cognitive behavioural treatment (ICBT) is chosen as comparator because its efficacy for adolescent depression has been demonstrated in previous randomized controlled trials. Hence, the experimental treatment, IPDT, is compared to an intervention with established efficacy, ICBT. The main hypothesis is that IPDT will demonstrate non-inferiority to ICBT in reducing depressive symptoms in adolescents with MDD.

### Intervention description {11a}

Both interventions consist of eight therapist-supported self-help modules delivered over 10 weeks on a secure online platform [25]. The modules contain text and videos followed by assignments the patients send to their therapist, receiving feedback within a few days. In addition, participants in both arms of the study will receive 30 min of weekly therapist support via text chat, which was found to be an important ingredient in the empirically supported version of ICBT for adolescents [9, 10]. A guidance protocol (available upon request) has been developed for contact with participants, message frequency and support for handling participants who do not follow their treatment protocols. This guidance protocol is being applied to both treatments to ensure the amount of contact and guidance is comparable in both interventions and further, to ensure the differences amongst the treatments solely regard content and not format.

#### IPDT

The IPDT programme was developed specifically for this project and tested in a pilot RCT [23]. It is based on similar psychodynamic principles as an Internet-based treatment with demonstrated efficacy amongst adults, but adapted to be suitable to adolescents [19, 20, 22]. Through text, videos and a series of experiential exercises, participants are taught how emotional conflicts may underlie and maintain depressive symptoms, how to notice their own anxiety and emotional avoidance (defenses) and how to approach previously warded off feelings. The final part of the programme contains material on how one may express previously warded off emotions to improve important relationships.

#### ICBT

The ICBT programme has previously been evaluated for adolescents suffering from depression [9, 10]. The modules target behavioural and cognitive factors documented to reduce symptoms of depression and anxiety. The treatment programme contains psychoeducation, behavioural activation, cognitive restructuring, affect regulation, anxiety management, and relapse prevention.

### Criteria for discontinuing or modifying allocated interventions {11b}

Criteria for discontinuing is severe psychiatric deterioration during treatment (such as severe suicidality or onset of psychosis) or severe social adversity (such as maltreatment in the family). When there is an indication of such adverse events, the PI (an experienced clinical psychologist) will conduct an assessment over telephone with the adolescent and, if indicated, the parents. The project psychiatrist could also be consulted in these cases. If the participant’s psychiatric deterioration is severe, he/she will be referred to psychiatric care. If the participant is the subject of maltreatment safeguarding standard operating procedures will be followed, including immediately and in accordance with Swedish law reporting this to the local social services for further action.

Allocated interventions could be modified if a participant lacks energy to fulfil all components of the treatment programme. For example, an agreement could be made with the participant to read less of the self-help material or do fewer of the exercises, compared to the full treatment programme. Participants adherence and activity within treatment, i.e. treatment dose, is automatically registered on the treatment platform and will be reported in the trial paper.

### Strategies to improve adherence to interventions {11c}

All participant activity in the programme, such as logging in on the platform, reading modules, completing exercises and attending chats, is automatically recorded on the platform and can thus be monitored and subsequently reported. Participants who miss chat sessions in either arm of the study will receive a text message immediately encouraging them to book a new chat session. Participants that have not had any contact with their study therapist for a full treatment week (i.e. neither completing exercises, writing messages nor attending the chat session) will be contacted by their therapist, initially by text message, thereafter by a phone call. If a participant keeps being nonresponsive for several weeks during the treatment, they will still receive a weekly message from their study therapist.

### Relevant concomitant care permitted or prohibited during the trial {11d}

No concurrent psychological treatment is permitted in order to be included in the trial. Participants are informed that they should not enter concurrent treatment during the treatment phase of the trial. However, if they do so, this is not cause for exclusion from the trial. Participants are asked at post-treatment and all follow-up about any concurrent treatments and this is carefully recorded. Psychotropic medications during the period of treatment are permitted, granted that treatment dose have been stable for at least 1 month prior to enrollment, with no planned dose adjustments during the trial.

### Provisions for post-trial care {30}

If a participant suffers from substantial psychiatric problems at treatment termination or follow-up, he/she will be offered referral to post-trial care at a psychiatric outpatient unit. Any participants who suffer harm from trial participation will be eligible for compensation in line with the rules of Stockholm University’s insurance for research participants at the university (an insurance called “Särskilt personskadeskydd”).

### Participant timeline {13}

**Fig. 1:**
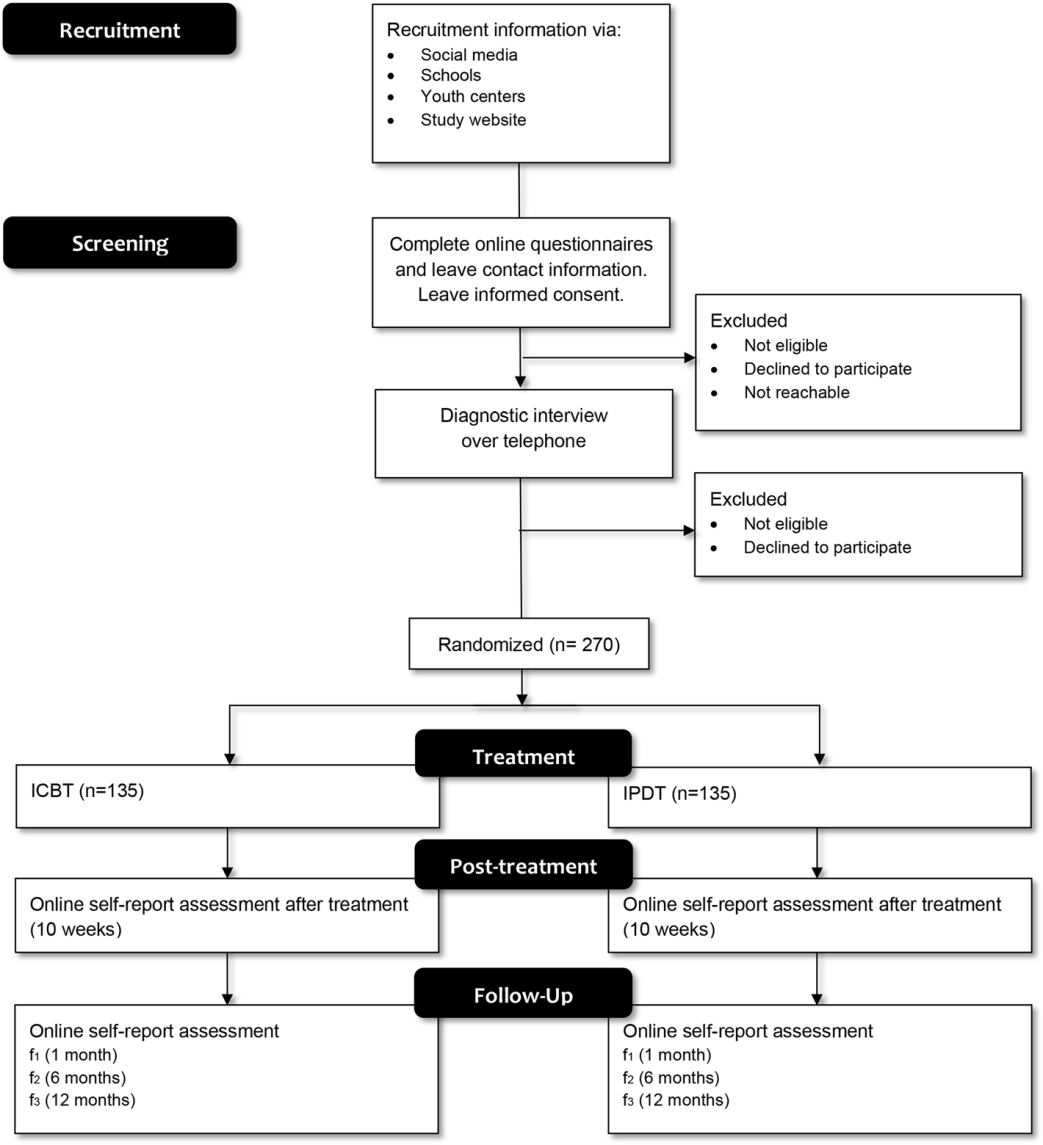
Consort diagram

See Fig. 1 for a CONSORT diagram of the participant timeline. Participants interested in the project will be directed to an online website where they can access further information about and register for the project, thereby acquiring access to the screening forms for the trial. If fulfilling criteria, and not fulfilling exclusion criteria according to the screening forms, a diagnostic telephone interview will be held to further establish a participant’s fulfilment of the inclusion criteria and non-fulfilment of any exclusion criteria. Eligible participants will be asked to confirm their participation in the study, by electronic written informed consent, thereafter they will be randomized and allocated to treatment.

### Sample size {14}

In order to assess non-inferiority and superiority using a repeated-measures design, power calculations for two-level LMMs were made following Galbraith and Marschner [29] using the R-package powerlmm v. 0.4. A non-inferiority bound of *d* = 0.30 was set alongside an α at 0.05, a variance ratio of 0.75, an attrition rate of 20% and intermittent missing data on weekly measures of 20%. Based on these calculations, a total sample size of 270 is needed to reach 80% power. The power analysis is based on data from the pilot trial [23]. The non-inferiority margin was based on earlier non-inferiority studies comparing face-to-face PDT to CBT in adults suffering from depression, in which Cohen’s *d* = 0.30 was considered as a minimal clinically meaningful difference. Driessen et al. [30] defined non-inferiority as Cohen’s *d* = 0.30 for all continuous outcome measures. Connolly Gibbons et al. [31] defined non-inferiority as 2.5 points on the Hamilton Depression Rating Scale, which was equivalent to a Cohen’s *d* of 0.29.

### Recruitment {15}

Participants will be recruited primarily by advertisements on social media. In addition, junior and senior high schools as well as healthcare providers, youth clubs, social workers and similar organizations will be contacted with information about the study. User organizations will be involved in informing such organizations about the study and recruiting participants.

## Assignment of interventions: allocation

### Sequence generation {16a}

Eligible participants will be randomized to one of the two arms (1:1 ratio). An independent researcher who is not involved in the study will conduct the randomization procedure via a computerized random number service (random.org). Randomization will be done in blocks of various sizes pending on the amount of adololescents found eligible at each inclusion conference during the project reqruitment period.

### Concealment mechanism {16b}

Randomization takes place after the participants have been found eligible through screening and subsequent diagnostic interviews, have been invited to participate in the study and confirmed their informed decision to participate. Subsequently, an independent researcher who only has access to a list of anonymous ID codes conducts the randomization. Thus, the randomization is conducted after completion of all baseline measures and final enrolment. The randomization is conducted in two steps. First, the independent researcher randomizes the two conditions IPDT and ICBT to decide what treatment should be allocated to participants on the first half of the list and what treatment should be allocated to participants on the second half of the list. Second, the anonymous ID codes are randomized, leading to a randomization list.

### Implementation {16c}

Allocation sequence is generated by the independent researcher through randomization. This will subsequently be sent to the PI, deputy PI and all project coordinators. Project coordinators will then assign the participants to interventions in accordance to the randomization list. Within hours of randomization, the study therapists are informed who will be treating which participant. The treatment begins the following day.

## Assignment of interventions: blinding

### Who will be blinded {17a}

Due to the nature of psychological treatment trials, neither participants nor therapists can be blind to treatment allocation, and all the outcome measures are self-rating scales, thereby rendering the blinding of assessors irrelevant. Prior to randomization, eligible participants are informed that they will be randomized to one out of two treatments, but information about the treatments is limited to format such as duration, structure (i.e. reading texts, therapist support via messages and chat sessions, the guided self-help format), and the hypothesis of the trial, i.e. that the treatments do not differ in effect for depression. Any acronyms or further characteristics of the specific treatments are avoided, in order to avoid expectancy effects related to prior knowledge or assumptions regarding specific treatments after randomization.

For the statistical analysis, groups will be masked meaning that the data analysts will be blinded. This means that two non-inferiority tests will be run, comparing each group’s non-inferiority towards the other, but only the one assessing non-inferiority for IPDT against ICBT will be retained.

### Procedure for unblinding if needed {17b}

Not applicable. The participants’ allocation to intervention is not blinded for the study therapists, the project coordinators or the PI.

## Data collection and management

### Outcomes {12}

The primary outcome will be severity of depressive symptoms, defined as the difference between groups in estimated change in depressive severity from baseline to end of treatment (10 weeks). Secondary outcomes will be anxiety symptoms, emotion regulation and self-compassion at treatment termination. In addition, long-term outcome in depressive and anxiety symtoms is measured at follow-up up to 1 year after termination. See the section “Data collection and management” below for details about the instruments used for measuring these outcomes.

### Plans for assessment and collection of outcomes {18a}

Data will be collected at baseline, weekly during treatment, at treatment termination as well as at three follow-up occasions (1, 6 and 12 months after termination).

All instruments consist of online-administered self-report questionnaires with established validity and reliability. Four self-rating scales will be administered weekly to track the primary outcome and possible mediators, whilst other questionnaires will be solely administered at baseline, termination and follow-up (see Table 1 for the measurement timeline).

**Table 1:** Schedule of enrolment, interventions and assessments

Activity/assessment	Online screening/baseline	Telephone interview	Post-randomization		Follow-up		
	***− t***_**1**_	***t***_**0**_	***t***_**1–10**_ (weekly)	***t***_**11**_ (post)	***f***_**1**_	***f***_**2**_	***f***_**3**_
Informed consent	X						
Demographic data	X						
ICBT/IPDT			X				
M.I.N.I 7.0		X					
C-SSRS		X					
DSRFI		X					
Treatment Expectancy^a^	X		X				
QIDS-A17-SR	X		X	X	X	X	X
SAI-C/SAI-T			X				
GAD-7	X			X	X	X	X
SCS-SF	X			X			
PID-5-BF	X						
TiC-P	X						X
ERSQ-27	X			X			
ERSQ-9			X				
ECR-RS	X						
OPD-SQS	X						
Termination form				X			

*M.I.N.I* Mini International Neuropsychiatric Interview, *C-SSRS* Columbia Suicide Severity Rating Scale, *DSRFI* Depression-specific reflective functioning interview, *QIDS-A17-*SR Quick Inventory of Depressive Symptomatology in Adolescents, *SAI-C* Session Alliance Inventory – Client version, *SAI-T* Session Alliance Inventory – Therapist version, *GAD-7* Generalized Anxiety Disorder -7, *SCS-SF* Self-Compassion Scale short-form, *PID-5-BF* The Personality Inventory for DSM Short Form, *TiC-P* Trimbos and Institute of Medical Technology Assessment Cost Questionnaire for Psychiatry, *ERSQ* Emotion Regulation Skills Questionnaire (27/9 items), *ECR-RS* Experiences in Close Relationships - Relationship Structure*, OPD-SQS* OPD-Structure Questionnaire short version. *F*_1_: 1 month, *F*_2_: 6 months; *F*_3_: 12 months

^a^“How much do you expect your depression to improve as a result of treatment?” on a 7-point Likert scale ranging from “− 3” (I expect to feel much worse) to “3” (I expect to feel much better)

#### Primary outcome

The primary outcome will be depressive symtoms, measured with the Quick Inventory of Depressive Symptomatology in Adolescents Self-Rated (QIDS-A17-SR [32]). The QIDS-A17-SR is a self-rated measure which has shown reliability and validity in previous studies amongst both adults and adolescents (e.g. [32–34]). Its brevity allows for weekly symptom ratings, which is needed for more sophisticated longitudinal statistical analyses. Assessments will be made via Internet-delivered self-rating forms pre-treatment, weekly during treatment, post-treatment and during follow-ups. The adolescent version is identical to the adult version with the addition of simultaneously assessing increased irritability as a symptom of adolescent depression.

#### Secondary outcomes

All secondary outcome measures have shown reliability and validity in previous studies. The secondary outcome measure for anxiety symptoms will be the Generalized Anxiety Disorder 7-item scale (GAD-7 [35]). The Emotion Regulation Skills Questionnaire (ERSQ [36]) will be employed to evaluate whether or not treatments are associated with participants’ enhanced capacity for emotion regulation. To assess their capacity for self-compassion, we will apply the Self-Compassion Scale Short Form (SCS-SF [37]).

#### Additional instruments

This trial also encompasses a range of measures to be analysed in additional papers following the main outcome paper. We will employ a range of measures to assess possible moderators for treatment effects: attachment, measured with the Experience in Close Relationships – Relationships Structure (ECR-RS [38]); self compassion, measured with the SCS-SF [37]; personality structure, measured with the OPD-Structure Questionnaire Short form (OPD-SQS [39]); and personality problems, measured with the Personality Inventory for DSM short form (PID-5-BF [40, 41]), all of which will be assessed at baseline.

We will also conduct a short interview at baseline to assess depression-specific reflective functioning (Mechler, Lindqvist, Falkenström & Möller: Depression-specific Reflective Functioning Interview for adolescents, Unpublished), which seems to work as a predictor for both the therapeutic alliance and outcome in standard face-to-face psychotherapy targeted at depression [42].

Possible outcome mediators will be assessed with the following instruments administered weekly during treatment: a nine-item version of the Emotion Regulations Skills Questionnaire (ERSQ-9 [36]), the Session Alliance Inventory (SAI [43]) measuring treatment alliance as experienced by both therapists and participants, and the single-item expectancy measure (adapted from Moras and Jones [44] and Connolly Gibbons et al. [45]). The SAI and expectancy measures will be filled out by participants and therapists alike.

Cost-effectiveness will be assessed with the Trimbos and Institute of Medical Technology Assessment Cost Questionnaire for Psychiatry (TIC-P [46]) at baseline and at the 12-month follow-up. Following recommendations for assessing cost-effectiveness in adolescents’ treatments [47], solely the sections of the TIC-P regarding healthcare use will be administered.

### Plans to promote participant retention and complete follow-up {18b}

Participants who discontinue the intervention will be offered the choice not to receive any additional weekly ratings, but will be asked to respond to post-treatment and at follow-up assessments. Hence, outcome data will be collected from the entire ITT sample at post-treatment and all follow-up points. Participants are allocated self-rating forms over the Internet and receive an email notification regarding this. Two and 4 days following the allocation, they receive automatic text message (SMS) reminders if they have not completed the forms. After this, participants that still have not completed their forms are contacted per SMS and phone by the study coordinators. No financial or material rewards are given to the participants for filling out the forms. However, at the follow-up assessment after 12 months, participants are informed that 100 SEK are given to charity organizations for each participant completing the follow-up measurements.

### Data management {19}

The study’s data management will follow the established procedures for studies on Internet-based treatments [48]. These procedures have been applied in a vast number of studies conducted over several years. All individuals who participate in the project will be assigned a unique ID that will be used throughout the project to communicate via the Internet. All correspondence with the participants will be made through a secure communication system; importantly, no communication with participants will occur via standard email. All communication and data collected through Internet-based self-report measures will be exclusively associated with each participant’s unique ID, and no personal information shall be discussed. During the project, all data (i.e. communication and data from self-report measures) will be stored in an encrypted database. Access to this database is controlled by the computer system so that the PI and the study coordinators have complete access to view. No one can change the values in the database and everything is logged. Personal information will be stored in a fireproof safe that solely the study administrators can reach. The key that links participants to their unique IDs will be stored in a separate fireproof safe to fulfil the regulations concerning safe data storage, and the data management procedures adhere to GDPR.

### Confidentiality {27}

All data are kept confidential through automatically generated study codes that are given to each participant. Data about a certain participant on the Internet platform and in the data base will solely use this study code as the identification. The identification key that connects the participant’s study code to the actual personal information (full name, Swedish personal identification number, and address) is stored in a fireproof safe that solely the study administrators can reach.

### Plans for collection, laboratory evaluation and storage of biological specimens for genetic or molecular analysis in this trial/future use {33}

Not applicable. No biological specimens will be collected.

## Statistical methods

### Statistical methods for primary and secondary outcomes {20a}

Statistical reporting will follow the CONSORT standards [49]. All participants who are randomly assigned to a condition shall be entered into the main analysis (intent-to-treat analysis = ITT) of the primary outcome measure (QIDS-A17-SR) at the primary assessment point of treatment termination (10 weeks). A secondary analysis of the primary outcome measure will include a per-protocol analysis.

In order to fully explore trajectories of change, a multilevel growth curve level strategy [50] will be employed for measures assessed weekly, comparing estimated change in depressive symptom severity from baseline to end of treatment between groups. Differences in efficacy between conditions will be investigated by modelling interaction effects of group and time. These methods have been recommended for RCTs that investigate Internet interventions [51]. One important advantage is their ability to handle missing data using a maximum likelihood estimation [52]. Non-inferiority will be defined as fulfilled when the upper 90% confidence interval of the estimated QIDS-A17-SR for the IPDT group at treatment termination is below the estimate for the ICBT group plus *d* = 0.30 (i.e. the non-inferiority margin).

The number of patients who changed reliably, as estimated by the Reliable Change Index (RCI [53]), will be reported to provide an estimate of improvement/deterioration in each group that is not attributable to chance. Response will be defined as reliable improvement (i.e. improved more than the RCI), whilst also scoring at least 2 SD below the pretreatment mean. Partial response will be defined as fulfilling the RCI whilst scoring less than 2 SD below the pretreatment mean. Remission will be defined as scoring 6 or below on the QIDS-A17-SR [33].

Secondary outcomes will be analysed primarily according to ITT and with treatment termination as primary assessment point. Mean scores at termination will be compared across groups using analysis of covariance (ANCOVA) controlling for baseline scores.

### Interim analyses {21b}

No interim analyses will be conducted. However, analyses of attrition will be conducted in order to increase the sample size in case of unexpectedly high attrition from weekly or post-treatment assessments.

### Methods for additional analyses (e.g. subgroup analyses) {20b}

No subgroup analyses are planned for the main outcome paper. Instead, moderator analyses will be conducted at a later stage (subsequent to the main outcome paper) to analyse whether certain factors predict differential suitability to the interventions.

### Methods in analysis to handle protocol non-adherence and any statistical methods to handle missing data {20c}

All primary analyses will be conducted according to ITT, using linear mixed modelling (LMM). LMM handles missing data with maximum likelihood estimation. Secondary analyses of the primary outcome measure will include a per-protocol analysis (PPA, which includes all participants who adhered to the protocol). Per protocol analyses will include completers, defined as the combination of having completed at least five modules (defined as completing at least one exercise per module), having attending at least five chat sessions and completing the post-treatment assessment.

## Oversight and monitoring

### Composition of the coordinating centre and trial steering committee {5d}

The Trial Steering Committee (TSC) will be composed of all investigators (authors). The TSC will meet according to key milestones and will have responsibility for project oversight, meeting key milestones, methodological and statistical conduct of the research, reporting to funder, review of risks and issues and ensuring the scientific integrity of the protocol and conduct of the study.

### Composition of the data monitoring committee, its role and reporting structure {21a}

The Data Monitoring Committee is composed of an independent researcher, an administrator and a clinician not otherwise involved in the study. The primary aim of the DMC will be to monitor progress of the study (e.g. reviewing recruitment and retention rates) and to review participant safety; all serious adverse events (SAEs) will be reported to and reviewed by the DMC as will any participant experiencing substantial psychiatric problems at treatment termination. The DMC will also make periodic checks of data completeness and accuracy.

### Adverse event reporting and harms {22}

All SAEs (e.g. suicide risk, substance misuse, ongoing maltreatment, or sexual abuse) during treatment will be registered by the principal investigator and the DMC. In addition, as suggested by Rozental et al [54], any negative effects will be monitored and reported. At post-assessment in a questionnaire with open questions, the participants will be urged to express their thoughts and feelings about their respective treatments and therapists as well as describe any adverse experiences whilst under treatment.

### Frequency and plans for auditing trial conduct {23}

Stockholm University does not have a specific unit for auditing trial conduct. The research group contains members both with allegiance to PDT and CBT, respectively, to ensure that both treatments are conducted in an adequate way. An independent researcher, external to the study’s project group, will be present and monitor the procedures as the database is extracted from the Internet platform and masked with regard to treatment arm prior to the outcome analysis.

### Plans for communicating important protocol amendments to relevant parties (e.g. trial participants, ethical committees) {25}

It is highly unlikely that important protocol modifications will occur. If we should consider any such modification, we will first have to apply to the Swedish Ethical Review Authority for permission to undertake these modifications. If modifications are undertaken, they will be reported to the trial registry ISRCTN. Changes to the protocol will also be reported in publications of the study’s findings.

### Dissemination plans {31a}

The primary outcome paper will be open-access and present outcome data in a peer-reviewed journal. No outcome data will be published or presented before the data collection process is completed. Subsequently, the results will also be presented at scientific conferences and in popular science texts that are comprehensible for a wider public. A popular science summary of the results will be posted online for laymen and study participants.

## Discussion

The need to develop early, brief, focused and effective interventions for depressed adolescents is urgent. Internet-based interventions are designed to reach, amongst others, people who do not access youth mental health services, or seek or receive traditional psychotherapy. One aim is to increase their access to psychological treatments, as well as increase such treatments’ cost effectiveness. Making treatment more easily accessible might also propose major health benefits because accessible treatment enables adolescents to apply for and receive treatment at an earlier stage of a psychiatric disorder. Internet-based interventions also have the potential to reach patients who would otherwise avoid seeking treatment due to social stigma [55]. It has been suggested that Internet-delivered treatment might also be useful for reaching adolescents with more severe symptoms who are reluctant to seek care on their own [56].

Empirical support has already been established for ICBT and the ICBT protocol used in the present study achieved clinical significant change in 46% of cases [10]. Preliminary results from the pilot IPDT trial suggest similar response rates. The trial is based on an extensive power analysis, based on data from two previous trials as well as a pilot trial, and especially taking the specific statistical analyses that are planned (i.e. multilevel modelling) into account. This trial is designed to investigate whether or not IPDT is non-inferior to ICBT with regard to depressive symptoms as well as determine whether or not the treatments are comparable regarding cost-effectiveness, thereby increasing treatment alternatives. Several secondary outcome measures are also assessed, in order to investigate possible differential effects of the treatments. Furthermore, we hope that the future moderator analyses will provide information regarding which adolescents will benefit the most from ICBT and IPDT, whilst mediator analyses will shed light on possible different treatment trajectories and processes.

This study compares two active treatments based on the hypothesis that they do not differ in effect. Conditions are matched in time, frequency and amount of contact, thereby reducing possible effects of such structural factors. One limitation is that we do not have an inactive control group to adjust for possible spontaneous remissions or regression to the mean, although both treatments have been previously tested against inactive control conditions with large effects. Furthermore, inactive control conditions for depressed children are ethically difficult to justify, which is why we wanted to avoid their inclusion in the present trial. In addition, waiting lists are not recommended as comparison groups [57].

The inclusion of measures of several potential moderators and mediators allows for an investigation of various pathways of change in the two treatments as well as an exploration of the possible factors that influence “what works for whom”. Increasing treatment alternatives is of utmost importance as response rates suggest that a substantial proportion of patients do not respond to existing, evidence-based treatments. In the future, if IPDT would prove non-inferior compared to ICBT, it would be relevant to conduct a cross-over-study, investigating whether patients not helped by one of the treatments would be helped by the other. The use of a guidance protocol ensures that conditions are matched in terms of guidance and amount of contact. Adverse events shall be rigorously tracked, and participants will be asked about any negative effects that result from their treatments.

This trial is the first to compare the effects of IPDT on adolescent depression to those of another active treatment. This trial’s results will be significant for expanding the range of time- and cost-efficient treatment alternatives to adolescents who suffer from depression, a group who immensely needs such attention.

## Trial status

Recruitment for the trial commenced in August 28, 2019, and is planned to extend into October 2020.

## Data Availability

The datasets generated and/or analysed during the current study are not publicly available due to the sensitive nature of the data collected from adolescents about mental health but will be available upon reasonable request. Access is given to the full protocol by this publication. The dataset will be available upon reasonable request.

## Abbreviations

CBT: Cognitive behavioural therapy
CONSORT: Consolidated standards of reporting trials
ICBT: Internet-based cognitive behavioural therapy
IPDT: Internet-based psychodynamic therapy
ITT: Intent to treat
LMM: Linear mixed modelling
MDD: Major depressive disorder
PDT: Psychodynamic therapy
PI: Principal investigator
PPA: Per protocol analysis
RCI: Reliable change index
RCT: Randomized controlled trial
SAE: Serious adverse event
